# Understanding Marketing Responses to a Tax on Sugary Drinks: A Qualitative Interview Study in the United Kingdom, 2019

**DOI:** 10.34172/ijhpm.2022.5465

**Published:** 2022-02-23

**Authors:** Hannah Forde, Tarra L. Penney, Martin White, Louis Levy, Felix Greaves, Jean Adams

**Affiliations:** ^1^MRC Epidemiology Unit, University of Cambridge School of Clinical Medicine, Institute of Metabolic Science, Cambridge, UK.; ^2^Global Food Systems and Policy Research, School of Global Health, Faculty of Health, York University, Toronto, ON, Canada.; ^3^The Faculty of Health and Social Care, University of Chester, Chester, UK.; ^4^Public Health England, London, UK.; ^5^Department of Primary Care and Public Health, School of Public Health, Imperial College London, London, UK.; ^6^National Institute for Health and Care Excellence, London, UK.

**Keywords:** Sugar-Sweetened Beverages, Soft Drinks, Advertising, Taxes, United Kingdom

## Abstract

**Background:** The World Health Organization (WHO) recommends that countries implement fiscal policies to reduce the health impacts of sugary drinks. Few studies have fully examined the responses of industry to these policies, and whether they support or undermine health benefits of sugary drinks taxes. We aimed to explore the changes that sugary drinks companies may make to their marketing, and underlying decision-making processes, in response to such a tax.

**Methods:** Following introduction of the UK Soft Drinks Industry Levy (SDIL) in 2018, we undertook one-to-one semi-structured interviews with UK stakeholders with experience of the strategic decision-making or marketing of soft drinks companies. We purposively recruited interviewees using seed and snowball sampling. We conducted telephone interviews with 6 representatives from each of industry, academia and civil society (total n=18), which were transcribed verbatim and thematically analysed. Four transcripts were double-coded, three were excluded from initial coding to allow comparison; and findings were checked by interviewees.

**Results:** Themes were organised into a theoretical framework that reveals a cyclical, iterative and ongoing process of soft drinks company marketing decision-making, which was accelerated by the SDIL. Decisions about marketing affect a product’s position, or niche, in the market and were primarily intended to maintain profits. A product’s position is enacted through various marketing activities including reformulation and price variation, and non-marketing activities like lobbying. A soft drinks company’s selection of marketing activities appeared to be influenced by their internal context, such as brand strength, and external context, such as consumer trends and policy. For example, a company with low brand strength and an awareness of trends for reducing sugar consumption may be more likely to reformulate to lower-sugar alternatives.

**Conclusion:** The theoretical framework suggests that marketing responses following the SDIL were coordinated and context-dependent, potentially explaining observed heterogeneity in responses across the industry.

## Background

**Key Messages**

** Implications for policy makers**

- How companies respond to dietary public health policies like the UK Soft Drinks Industry Levy (SDIL) (a sugary drinks tax) may influence how effective those policies are.
- Soft drinks companies changed their marketing in response to the SDIL in a variety of ways including reformulation, brand acquisition, and changing packaging.
- A company’s marketing response to an intervention like the SDIL appears highly dependent on contextual factors such as brand strength, reputation, and the size of their portfolio, and so may be predictable.
- Policy-makers may want to consider these potential responses in designing more nuanced sugary drinks taxes to ensure that they maximise the potential effects of these interventions.

** Implications for the public**

Consuming less sugar could help people live healthier and longer lives. To help people consume less sugar, governments can tax sugary drinks. Such taxes might lead sugary drinks companies to increase the price of their drinks or make them less sugary and so lead to reduced sugar consumption. However, there are other ways companies could respond that might have the opposite effect on sugar consumption. We consulted with experts to explore the range of reactions that companies could take following a sugary drinks tax like the UK Soft Drinks Industry Levy (SDIL). We found that companies with certain characteristics were more likely to take specific actions in response to a tax. Knowing this means that other governments could design taxes in ways that are most supportive of companies responding to taxes in ways that will minimise how much sugar people consume.

Sugar consumption, particularly in liquid form, is an independent risk factor for non-communicable diseases like type II diabetes and heart disease.^1^ The World Health Organization (WHO) identifies the taxation of sugary drinks as a “best buy” to address non-communicable diseases.^2^ Sugary drinks taxes can reduce sugary drinks purchases,^3^ and whilst they might impact on product prices,^4^ this can be in category specific ways, rather than resulting in straightforward increase. There also remain unanswered questions about their mechanism of action and optimal design.^5^

Some studies have found that despite relatively acute price changes, the impact of sugary drinks taxes on purchases develop and evolve over time,^6^ suggesting price increases are not the sole mechanism of action. Sugary drinks taxes are hypothesised to prompt multiple changes.^7^ Some of these ‘spillover’ effects are likely to reinforce the public health purposes of these taxes (eg, signalling to citizens that sugary drinks are harmful), while others may undermine them (eg, companies increasing advertising to offset any reduction in sales). Better understanding of what spillover effects occur in what context could inform better tax design.^8,9^

Many of these spillover effects may involve changes in products, prices, promotion or placement,^7^ broadly understood as the ‘four Ps,’ or “marketing” mix.^10^ Marketing is defined as, “the activity, set of institutions and processes for creating, communicating, delivering and exchanging offerings that have value for customers, clients, partners, and society at large.”^11^ There is substantial evidence that food marketing can lead to changes in preferences, choices and consumption.^12^ Though less evidence focuses specifically on soft drinks marketing, an emerging literature confirms a similar link.^13,14^ While evaluations of sugary drinks taxes have focused on product sugar content and price changes,^15^ and some emerging evidence explores changes to advertisements,^16^we are not aware of studies that have explored changes in ‘marketing’ across the four Ps of the marketing mix following a sugary drinks tax.

One potential reason for little evidence about marketing changes may be the absence of clear understanding of the decision-making processes involved. Using the UK Soft Drinks Industry Levy (SDIL) (Box 1), we aimed to explore how and why marketing changes following a sugary drinks tax.

**Box 1. Study Context: The UK Soft Drinks Industry Levy**

** The UK Soft Drinks Industry Levy**

Two years after it was first announced, the UK government introduced the SDIL in April 2018, with the aim of encouraging reduced portion sizes and reformulation of high sugar drinks to lower sugar alternatives.^17^ The SDIL is a tiered tax on soft drink manufacturers and importers according to drink sugar content: £0.18/L for drinks containing >5 g/100 mL and ≤8 g/100 mL of added sugar, and £0.24/L for drinks with >8 g/100 mL of added sugar.

Evidence of the soft drinks industry’s response to the SDIL has emerged. Though consumption of drinks in the high levy tier was already falling prior to the announcement of the SDIL,^18^ the implementation of the SDIL was associated with increased prices, changes in product size, and change in the availability of high levy tier soft drinks.^19^ The announcement and introduction of the SDIL had no long term impact on soft drinks company share prices.^20^ The amount of sugar in soft drinks purchased also decreased by 30% between 2015 and 2018.^21^

----------------

Abbreviation: SDIL, Soft Drinks Industry Levy.

## Methods

We undertook one-to-one, semi-structured qualitative telephone interviews with stakeholders from academia, civil society, and industry. Interview transcripts were thematically analysed to develop a theoretical framework that shows how soft drinks companies might react to taxation. We situated the study in a pragmatist epistemological approach,^22^ accepting there may be multiple realities for soft drinks companies and given our focus on solving practical problems. Reporting adheres to the Consolidated Criteria for Qualitative Research (Supplementary file 1).^23^

###  Interviewees and Recruitment

Recruitment and data collection took place in January to May 2019. To inform recruitment and interviews, authors HF and TP theorised ways that soft drinks ‘companies’ [hereafter used to describe any producer or retailer of sugary drinks responsible for product marketing] might change marketing in response to the SDIL, using their knowledge of relevant literature. Ideas were grouped into a simplified, hypothesised process of marketing change: we anticipated the SDIL would prompt industry strategic decision-making followed by the selection and implementation of marketing activities (Supplementary file 2). This informed our participant inclusion criteria, which were: individuals with self-described first- (eg, works in marketing for a company) or second-hand experience (eg, studies company marketing) of soft drinks company decision-making or marketing.

 We purposively sought views across three sectors likely to have different, relevant perspectives: academia, civil society, and industry. ‘Industry’ included any individual with recent or current experience of producing, retailing, or marketing soft drinks; academics from public health, policy, and marketing fields were included; civil society representatives were identified based on experience with policy proposals for diet and obesity.

We employed the concept of inductive thematic saturation to determine the total sample size.^24^ We decided *a priori *to complete 15-25 interviews with equal representation across the groups – that is, 5-9 in each of academia, civil society and industry. As data collection and analysis advanced, we determined a total sample size of 18 with six in each group to approximate saturation, as there were mounting instances of the same codes and no new ones developing.^25^

Interviewees were iteratively recruited using seed and snowball sampling. The seed list consisted of the study teams existing contacts supplemented by searching for relevant organisations using internet searches and LinkedIn, a professional social media platform (https://www.linkedin.com/). Preliminary telephone calls and emails helped determine whether potential interviewees met inclusion criteria. Potential interviewees were sent an invitation and interviewee information sheet by email, with opportunity to ask questions before scheduling an interview. Reminder emails were sent if we received no response within two weeks of an initial invitation. Individuals were classified as “non-respondents” if they failed to reply in a further two weeks. To permit snowball sampling, interviewees were asked to share details of relevant contacts following their interview. Non-responses and invitation declines were recorded.

###  Data Collection

On the day preceding scheduled interviews, interviewees were sent a short briefing document on our hypothesised process of marketing change (Supplementary file 2) by email. Some academic interviewees were professionally known to the interviewer (HF). Interviewees were aware of the interviewer’s name and research interests: food industry and diet-related public health policies. It was assumed that interviewees were interviewed alone, though this was not objectively verified.

Interviews followed a semi-structured interview guide (Supplementary file 3). The guide was piloted in the first two interviews,^26^ prompting only minor amendments, and pilot interviews were subsumed into the main analysis. Interviews were audio-recorded and transcribed verbatim, and brief field notes made to aid interpretation. We expected interviews to last 45-60 minutes.

###  Analysis

**Figure 1:**
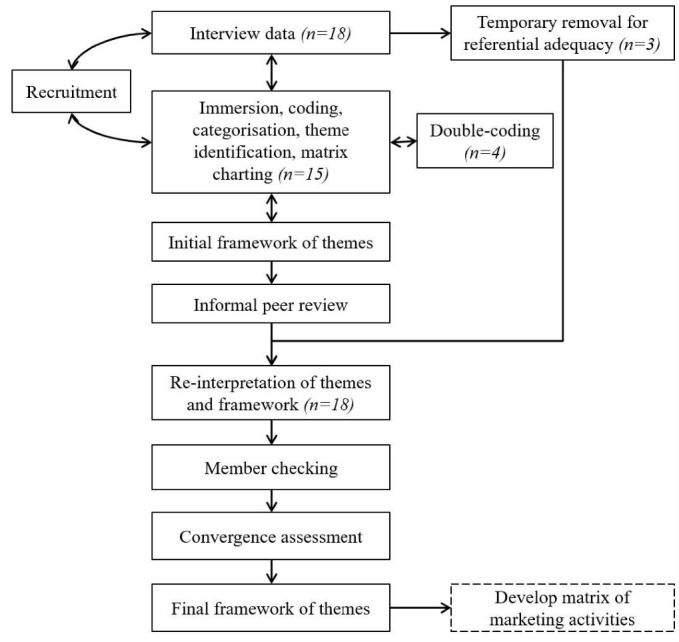
Summary of Data Analysis Procedure.

Analysis drew on the Framework method and is summarised in Figure 1.^27^ The hypothesised process of marketing change formed a basis for codes. By searching for relationships between themes, we developed a theoretical framework.

Data analysis began alongside interviews in four iterative, non-exclusive steps: immersion, coding, creating categories, and identifying themes.^28^ As we were seeking elaboration and clarification of our hypothesised process, analyses melded inductive and deductive reasoning.^29^ HF became immersed and familiar with the data by reading and re-reading transcripts. Descriptive labels, or ‘codes,’ were applied to transcript segments that appeared to address our research interests,^28^ and broadly grouped into sections depicted in our hypothesised process (Supplementary file 2): industry decision-making or manifestations of marketing change. Categories were created when similarities between codes were identified, from which we sought explanations and interpretations to identify themes. Themes are interpretative concepts that describe or explain parts of the data.^27^

HF, MW and JA then considered possible relationships between themes with reference to the original hypothesised process of marketing change (Supplementary file 2), to produce a revised theoretical framework. With reference to the Framework method, data were charted in a matrix that facilitated comparison between interviewee groups, culminating in an adapted convergence coding assessment (ie, a means of tracking the nature of contribution from each group).^30^ To support the generation of creative insights,^31^ particularly for visualisation, we used a combination of digital tools (Microsoft Excel; NVivo, version 12, QSR, Southport, UK) and whiteboard diagramming.

HF, who has experience conducting and analysing qualitative research,^32^ developed initial themes. A subset of transcripts (n = 4) were double-coded (MW = 2, JA = 2) and discussed in a data clinic.^33^ Referential adequacy was achieved by excluding three transcripts from initial coding.^34^ Several potential frameworks were discussed with the research team (TP, LL, FG) and a wider research group prompting reinterpretation of some themes, after which the excluded transcripts were reintroduced to verify findings against ‘fresh data.’

Finally, member-checking entailed sending a summary of findings to interviewees by email to ask how well they reflected interviewees’ experiences and views (see Supplementary file 4).^35^ Reminder emails were sent after two weeks; we assumed interviewees had no further comments if we received no response within a further week. Comments were collated and discussed by the authors, prompting further minor amendments to the theoretical framework and themes. The comments also prompted us to develop a matrix of marketing activities, which we achieved by returning to the data to review more explicit connections between contextual factors and marketing activities.

## Results

 We report interviewee recruitment and characteristics, and present themes with illustrative interview quotations. We then use a matrix presentation and narrative to describe relationships between company context and marketing changes. Finally, we present the theoretical framework derived from the themes and relationships to depict the soft drinks company decision-making process. As described and justified below, the theoretical framework developed by thematic analysis of interviews represents a cyclical process of decision-making about marketing, influenced by a company’s context and accelerated, but not precipitated, by the SDIL.

###  Interviewee Recruitment and Characteristics 

We interviewed 6 professionals from academia (identified using the codes ACA01-ACA06 below), 6 from civil society (CIV07-CIV12), and 6 from industry (IND13-IND18). To preserve anonymity, we report a summary of interviewee characteristics for each group in Table 1. Interviews lasted between 20 and 51 minutes (mean: 33 minutes).

**Table 1:** Interviewee Recruitment

**Interviewees/Interviewee Numbers**	**First Contact, N**	**First Responses,** **No. (%)**	**Referrals, ** **No. (%)**	**Interviews, ** **No. (%)**	**Summary of Interviewee Characteristics**
Academia/ACA01- ACA06	10	10 (100)	0 (0)	6 (60)	Marketing researchers with experience of public health; public health and policy researchers with experience of marketing; marketing and strategy researchers.
Civil society/CIV07- CIV12	13	8 (62)	0 (0)	6 (46)	Experience in social marketing, food campaigning, researching sugary drinks taxes, consumer or policy advocacy for health.
Industry/IND13–IND18	70	17 (24)	2 (3)	6 (9)	CEO of a small soft drinks company; marketers for medium-size soft drinks companies; former employee of a large soft drinks company; marketing strategists.

*Note*: ‘First contact’: initial contact made by email, telephone, or LinkedIn to an organisation or individual; ‘First responses’: reply to initial contact from an organisation or individual; ‘referrals’: individuals successfully recruited into the study by snowball sampling; percentages are proportions of initial contact.

 Abbreviation: CEO, chief executive officer.

Table 2presents a description of themes and supporting categories with example quotations.Themes were robust to reintroducing the three transcripts archived for referential adequacy (CIV10, CIV12, IND18). When a summary of findings was sent to interviewees (see Supplementary file 4), 11 provided feedback (61%). In response, we developed a matrix to depict the relationships between company context and specific marketing activities (Table 3), alongside an accompanying narrative. Convergence assessment found no areas of dissonance between interviewee groups; areas of silence appeared to reflect interviewees’ relative expertise. We then provide an accompanying narrative to describe the changes in marketing detailed in Tables 2 and 3, and their relationship with company context.

**Table 2:** Description of Themes and Constituent Categories With Illustrative Quotations

**Theme Name**	**Description of Theme and Constituent Categories (in Bold)**	**Illustrative Quotations of Theme**
On-going monitoring	Companies use various tools to continually monitor the market, to identify opportunities and threats for achieving a competitive commercial advantage.	*“Horizon scanning…the trend that’s going to hit the in the next year, two years, three years, and how can I make a sustainable product portfolio” *[IND15].
Identify stimuli	Companies identify stimuli – like the SDIL – in their context that encourage an accelerated review of their products’ market position to capitalise on marketing opportunities.	*“When these kind of things come into play they’re not new news…the only change when the levy was announced was it gave us a deadline to complete that work” *[IND14].
Determine products’ market position	Careful, iterative decision-making, often involving cross-departmental input, informs a product’s position in the wider market.	*“If you imagine it like a mixing desk in an audio studio … how do I mix this to create the maximum return for my shareholders?” *[CIV07].
External context	Factors outside a company that may influence its market performance are monitored. These include **competitor activity**, as success depends on relative performance, and a stimulus may provide an opportunity to differentiate. **Consumer preferences** are important because they are bi-directly associated with purchasing. **Retailers** are influential because they negotiate and enact some marketing activities, including price promotions. **Suppliers** influence feasibility of reformulation and new product development. Perceptions of the direction that** policyandregulation** may take influences responses to stimuli.	*“It’s an iterative, circular process of organisations driving consumer wants, and these becoming consumer needs, and then organisations fulfilling consumer needs” *[CIV07]. *“If the retailers think that your price is going to stop people buying the product the retailers will negotiate very hard with you” *[ACA01]. *“It may be that the threat of [sugar company] putting their prices up holds more of a threat than the levy, in which case you keep the sugar and pay the levy because otherwise you’re going to get screwed” *[ACA01]. *“We had seen the introduction of that schools legislation [which restricted what products could be sold in schools] as being kind of almost a forbearing of what might come” *[IND14].
Internal context	Factors within a soft drinks company are monitored to inform marketing decisions. Marketing changes must be consistent with **brandidentity**. Higher **brandstrength** may lead to price increases and diversification, while lower brand strength may lead to reformulation. **Capacityandwillingness** within a company determines the feasibility of activities: larger companies have more resource but may be less agile. A company’s **concernforreputation** may mean they want to ‘do the right thing.’ **Learningfromexperiences **of responding to similar stimuli determines a companies’ optimism in their response to the latest stimuli. Companies with a **large portfolio **can spread risk, meaning they have less incentive to reformulate.	*“If you’ve spent decades telling people this is what the Real Thing tastes like, the harder it is to actually change” *[IND17]. *“It’s very much capacity-led…we can’t handle doing campaigns and new product development at the same time” *[IND14]. *“Having had that experience, you know, both industry and to a certain extent governments were like, oh okay…we know what to do now in these situations” *[CIV07]. *“If a brand has a kind of portfolio of products, they might have looked at, ‘well we allocate our marketing spend slightly differently…can we move them onto milk-based drinks or juice-based drinks?’” *[CIV12].
Marketing	Companies make changes across the marketing mix to position a product. Activities include **reformulation**, where companies reduce the sugar content of levy-eligible drinks to avoid paying the full levy cost. **Increasing the price** of products enables companies to pass [at least some] cost of the levy to consumers. Some companies will **develop or acquire new products**, perhaps because there are no existing consumer taste expectations. Companies could also **change messaging** in theirmarketing, developing messages about health, continuity, heritage, or choice. **Changing product packaging**, perhaps across brands in portfolio, can help communicate these messages. **Reducing portion size** may encounter less resistance than reformulation, though more easily achieved once smaller product sizes are normalised. **PR campaigns**, such as those focused on sport, could promote drinks and boost reputation. Though technically possible, **changing the distribution** and **changing the placement** of products is difficult to negotiate.	*“Don’t change your packaging, don’t do a press release, just quietly get rid of the sugar and nobody notices if you do it well” *[CIV10]. *“A lot of it has been buying up other companies that have mid or low-calorie beverages and adding them to their portfolio” *[CIV11]. *“Almost kind of de-risking it by putting all their brands on pretty much the same platform, and saying effectively, you choose” *[IND17]. *“Focusing on portion size is the right solution…the alternative, which is to tinker with the formula is a really bad idea” *[ACA06]. *“If you can’t push the problem away.. [then you could] invest in CSR type activities, public education activities, things which can give your company a healthier look”* [ACA05]. *“They always sell through retailers, and so how the retailers respond is very important” *[ACA06].
Non-marketing activity	Companies also use activities that do not directly affect the relationship between a product and prospective consumer to position their products in a market. This includes **framing** either themselves or the stimuli in a way that protects profits, and **lobbying**, possibly against further regulations; these are likely to contrast their communication to the public.	*“(Example) soft drinks don’t contribute to dental caries, the problem is it’s the parents who don’t supervise the children brushing their teeth”* [ACA05]. *“They undoubtedly would have argued against it or argued for a different approach” *[IND16].
Purchase soft drinks	Companies will coordinate their activities in a way that retains profit, since this is their overwhelming concern.	“The idea that this was some kind of financial catastrophe has proven very untrue” [CIV10].

Abbreviations: PR, public relation; CSR, corporate social responsibility; SDIL, Soft Drinks Industry Levy.

**Table 3:** Factors in a Soft Drinks Company’s Internal and External Context That Possibly Increase the Likelihood of a Marketing Response^a^

		**Marketing Response**								
		**Reformulation**	**Develop or Acquire New Products**	**Change Messaging**	**Increase Product Price**	**Reduce Portion Size**	**New PR Campaigns**	**Change Distribution**	**Change Placement**	**Change Packaging**
Factor in the external context	Competitor activity	If competitors reformulate	If competitors develop/acquire new products	-	If competitors increase price	If competitors reduce size	-	-	-	-
	Consumers’ preferences	If interested in health, not wary of artificial sweeteners	If interested in health, low-sugar variants likely	If interested in health, health messaging likely	If below the consumer psychological threshold	If smaller products considered acceptable	If interested in health, health campaigns likely	-	-	-
	Policy and regulation	If responded to policy before or think more will follow	If think similar policy will follow, low-sugar variants	If wary of policy, create ‘health’ and ‘choice’ messaging	-	-	-	-	-	-
	Influential retailers	-	-	-	If aligns with retailers’ goals	-	-	-	If aligns with retailers’ goals	-
	Influential suppliers	If sugar suppliers are not influential	-	-	If sugar suppliers are not influential	-	-	-	-	-
Factors in the internal context	Brand identity	If identity not tied to sugar	If identity tied to sugar	If identity tied to sugar, messaging likely to be about continuity, heritage	If identity tied to sugar, more likely than reformulation	If identity tied to sugar, more likely than reformulation	If identity tied to sugar, sport or choice campaign likely	-	-	-
	Brand strength	If a weak brand	If a strong brand	If a weak brand that is easier to change	If a strong or ‘luxury’ brand	If a strong brand	-	-	If a strong brand	-
	Capacity and willingness	Less agile, lack infrastructure or willingness	May be easier to acquire products than develop new ones	If sufficient resource to support new campaign	If internal dynamics are supportive	If sufficient infrastructure and willingness	If company has international presence	If company has international presence	-	If sufficient resource available
	Concern with reputation	If concerned with appearing responsible	-	If want to appear healthy, health messaging likely	-	-	If want to appear healthy, sport or health campaigns	-	-	-
	Learning from experience	If reformulated successfully before	-	-	-	-	-	-	-	-
	Size of portfolio	If no low-sugar variants in portfolio	If no low-sugar variants in portfolio	Choice messaging if low-sugar variants in portfolio	-	-	-	-	-	-

Abbreviation: PR, public relation. ^a^Read as: marketing response that is likely to happen if a contextual factor is present eg, reformulation is likely to happen if competitors reformulate. ‘-’ is used where no evidence was found.

###  Company Context and Marketing Responses Following the SDIL

 Interviewees agreed that companies would respond to the SDIL using a combination of different aspects of marketing:

“*[marketing] is very much a recipe and you don’t bake a cake and then think, “now we’ll add the sugar”; you work out the recipe first and then bake the cake…it doesn’t make any sense to make decisions about one without considering the others” *[ACA03].

Companies were reported to continuously evaluate factors within a company – their internal context – and factors outside of a company – external context. They use point-of-sale data, market research, and observing competitor activity at trade shows to facilitate this contextual evaluation. This informs their assessment of their performance, enabling them to both identify and respond to stimuli like the SDIL. Interviewees reported that the SDIL sped-up this iterative process of contextual evaluation and response: *“catalysts to me are things that are accelerating change…100% [the SDIL] is a catalyst” *[IND18].The motivation for changing marketing in response to context and stimuli is to retain profit: *“marketing will do whatever it takes in order to maintain the bottom line” *[ACA05].

####  Reformulation

The most extensively described marketing response to the SDIL was reformulation to reduce sugar content and avoid paying the levy. Whether a company could and would reformulate appeared to depend on several contextual factors. Brand strength, which is the perception and value invested in a brand, significantly influenced reformulation. Customers were described as less price-sensitive to stronger brands, preferring to pay a higher price than consume a reformulated product, particularly if the brand strength is strongly connected to consumers’ perception of taste. Reformulation was more feasible for brands whose identity is orientated around health, whereas interviewees reported that companies with an identity tied to sugar can receive ‘backlash’ to reformulation *(“sugar was the whole point of it” *[ACA02]).

 Consumer preferences, and particularly trends for healthiness and lower sugar products, were also described to facilitate reformulation:

“*There’s a big section of the market that is interested in health and reducing sugar, you could say that that’s influenced the market more than the SDIL” *[ACA02].

 Reformulation was thought to help protect companies’ reputations, who wanted to be seen to be ‘doing the right thing’ by consumers and policy-makers. However, interviewees also said that some companies were wary of replacing sugar with artificial sweeteners because of consumers’ fears about their health effects.

 A company’s capital also affected likelihood of reformulation: those with substantial physical production infrastructure were thought to be less able to reformulate and those with more financial capital more able to reformulate. Sugar suppliers were also said to affect a company’s likelihood of reformulation, as they could respond to reduced demand across the sector by raising prices, negating any financial benefits to companies of removing sugar to avoid the levy. Practical factors related to the size of a company were also reported to influence a soft drinks company’s ability to reformulate. Larger companies with portfolios consisting of both high and low sugar products were thought less likely to reformulate, as they already have lower sugar alternatives.

Interviewees noted that some reformulation pre-empted the SDIL announcement, but also explained that companies probably explored other options before reformulating (*“move through a range of backstops…never move immediately to the reformulation stage” *[ACA05]*)*. Interviewees also described a “last mover advantage”, with some companies waiting to observe the results of competitors’ reformulation before committing.

 Interviewees described how experience of successfully responding to other legislation may have reduced resistance to reformulation. One interviewee described how their company’s reformulation following school food legislation had prepared them for the SDIL [IND14].

####  Develop or Acquire New Products 

Another commonly discussed marketing response to the SDIL was portfolio expansion by developing or acquiring new, lower-sugar, products. Like reformulation, some of this diversification was thought to pre-date the SDIL and reflected a focus on protecting profits by both *“maintaining the [price-insensitive] heavy user” *[ACA05],of core brandswhile also selling lower-sugar alternatives to health conscious or price-sensitive consumers.

As with reformulation, an existing portfolio consisting of levy-exempt drinks was thought to result in less pressure for new product development or acquisition. Interviewees described a decreasing marginal return with increasing portfolio size, as large portfolios risk diluting the power of flagship products. Brand strength and identity also influenced the nature and likelihood of acquiring or developing new products. Interviewees thought it easier for companies to acquire than change existing products, if those products had a strong taste expectation among consumers: *“a combination of both the classic heritage premium product kind of thing together with introducing new brands” *[ACA02]. The nature of a company’s physical and financial capital may have also determined whether companies developed or acquired new products. For some, it may be quicker and less costly to acquire products than to develop new ones.

Perceptions about future policy and consumer trends also influenced diversification, with some companies thought to be ‘future-proofing’ their portfolio through expansion. One industry interviewee noted that the normalisation of lower-sugar drinks was leading some companies to search for alternative ways to identify their products as ‘healthy,’ adding vitamins, fibre or protein:* “‘no added sugar’ just isn’t cutting it for [consumers] anymore”* [IND14].

####  Change Messaging

 Some companies were reported to change the overall essence of their communications to consumers and policy-makers following the SDIL. These communications take place across the marketing mix. Though some companies chose to enact new messaging campaigns, it was noted that campaigns are often costly and so may only be a feasible for larger companies. One industry interviewee described how their company had to choose between developing a new product and launching a new messaging campaign [IND14].

 Frequently described marketing messages centred around health, continuity, heritage and choice. For those companies concerned about consumer preferences, reputation and health, their messaging was more likely to focus on health:

“*‘We’ve reformulated, we care,’ you know, ‘we want to help the obesity crisis,’ they can use that as a kind of social marketing campaign” *[ACA01].

 Given perceived concerns about the health impacts of artificial sweeteners, it was thought that messaging would be cautious about explicitly mentioning these. An interviewee also explained that brands are often marketed using multiple messages, for example a lead message (such as health) supported by one or two others (choice, heritage). Changing messaging may involve reprioritising these existing messages, rather than developing new messages.

Interviewees thought it would be harder for strong or unique brands to dramatically change their messaging without undermining the brand. In these cases, a focus on continuity and heritage that reassured consumers was considered preferable: *“protecting the heritage, protecting the kind of uniqueness of certain brands” *[ACA02].Whilst most messaging is brand-specific, some may take place across portfolios. Interviewees described how a company had unified the messaging across their portfolio, possibly to position both high and low sugar consumption as a consumer choice that their portfolio could accommodate.

####  Increase Product Price

 Increasing the price of levy-eligible products in response to the SDIL was considered more likely and successful for strong brands, as they have the least price-sensitive consumers. As with reformulation, interviewees also thought it possible that price changes could be subject to ‘domino effects,’ with a few companies having to blaze a trail before others rapidly followed suit.

The intermediary role of retailers was considered important in relation to brand strength and price increases: “*negotiating a price increase into the retail trade is a, it’s like a dance” *[CIV07]. Stronger brands were considered more able to negotiate lower margins with retailers because they are viewed as ‘commodity products’ that define ‘the soft drinks aisle.’ Retailers can demand higher margins from weaker brands because not selling these is unlikely to impact overall custom.

 Varying brand strength within a company’s portfolio, combined with negotiations with multiple retailers (who themselves are in competition), and company-specific calculations of margins, meant one interviewee thought there were practical difficulties in differentially pricing higher versus lower sugar drinks within and between companies. Differentially pricing high and low sugar variants within a brand was also thought to make running price promotions logistically challenging and harder to communicate to consumers. An interviewee thought this might lead companies with large portfolios of products with varying sugar content to maintain price uniformity across their portfolio [CIV07]. With many soft drinks companies operating internationally, these dynamics can play out at both a national and international level.

####  Reduce Portion Size

Reducing portion size was a further potential marketing response to the SDIL. Interviewees had seen this in response to previous legislation, such as menu labelling. Whist reducing portion size without reducing prices is equivalent to increasing price in volume terms, one interviewee thought reducing portion size was more acceptable to consumers than increasing price as it does not pass a *“psychological threshold” *[ACA06].

 As with price, interviewees suggested that reducing portion size is more acceptable to consumers of stronger brands than reformulation, particularly those with previous negative experiences of reformulation. Again, the importance of normalisation of, in this case, smaller pack formats across the sector following the SDIL was noted [IND15], reinforcing that some company-level reactions can be amplified by significant players to become a sector-wide strategy.

 Avoiding alerting consumers to smaller portion sizes was considered particularly important in ensuring the acceptability of this strategy. An interviewee reported that a company may have adopted this strategy if they were specifically measuring their performance by profits rather than volume sales.

####  Change Distribution, Placement and Packaging; New Public Relations Campaigns

 Though descriptions of other marketing activities were less salient, interviewees said it was possible that every marketing lever could have changed in response to the SDIL, including distribution, placement and packaging. Interviewees also explained that large multinational companies might have recouped lost UK sales following the SDIL by increasing sales elsewhere. As with price changes, retailers were thought to have been central in determining whether a company could reposition their products in retail outlets. The most desirable locations, such as end-of-aisle and eye-level shelves, usually cost more to occupy:

“*Soft drink and other food manufacturers pay the grocery stores very large fees in order to first get on the shelf, to stay on the shelf, to get at eye level, to get on the end of aisle displays, to get in the checkout, to really be more visible and promoted, to have these very aggressive sales” *[CIV11].

 Again, those products with higher brand strength or companies with more financial capital were thought more able to use this lever.

 Interviewees cited some brands they thought had changed their packaging in response to the levy. In some instances, repackaging lower sugar variants to resemble higher sugar ones more closely, may have normalised lower sugar drinks for consumers.

 Interviewees touched on the potential for using public relations (PR), particularly as part of Corporate Social Responsibility strategies in response to the SDIL, giving the example of physical activity promotion. As PR is usually expensive, interviewees thought only larger companies would have been able to use this lever.

###  Theoretical Framework 

**Figure 2:**
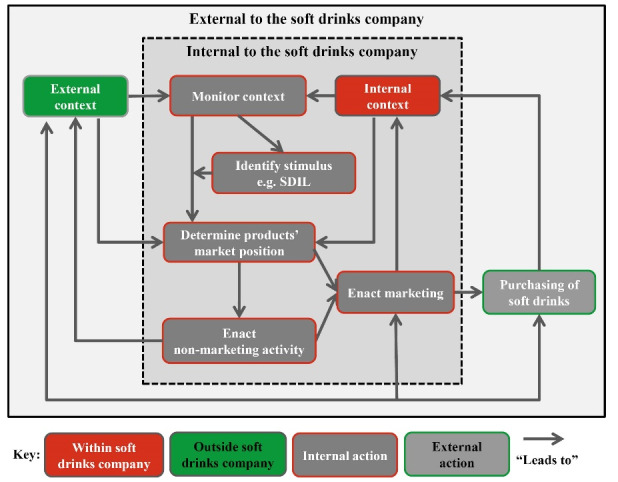
Theoretical Framework of Marketing Decision-Making in Response to the Soft Drinks Industry Levy. Abbreviation: SDIL, Soft Drinks Industry Levy.

Using the relationships between themes, we developed a theoretical framework. This shows a process of marketing decision-making in which a stimulus such as the SDIL accelerates, rather than precipitates, soft drinks companies’ to review a product’s position in the market (at a brand or company level) (Figure 2). Both marketing activities and non-marketing activities are used to determine a product’s market position, in turn influencing soft drink purchases and profits and so acting as a feedback loop. Though this process is similar for each company, the differing internal and external contexts of each company means the process results in a unique combination of marketing activities: *“each company will not go through the same decision …[ the outcome] depends on the company” *[ACA01].

## Discussion

###  Summary of Study Findings

 Using the SDIL as context, we explored how soft drinks marketing might change in response to a sugary drinks tax. We found that the underlying process of marketing decision making is continuous and iterative, and accelerated rather than precipitated by the SDIL. Possible marketing changes in response to the SDIL spanned the full suite of potential marketing activities, including changes to the product, its price, placement and promotion. The specific selection of marketing activities chosen by any particular company appears to be determined by context-specific internal and external factors. For example, companies with high- and low- sugar products in their brand portfolio appeared less likely to reformulate, while those with high brand strength, high-sugar drinks were more likely to increase prices. Understanding the role of context may help explain possible heterogeneity in marketing responses to the SDIL across the sector. This could help predict company-level marketing changes following sugary drinks taxes with implications for public health policy development.

###  Strengths and Limitations of the Study

The openness of the marketing process initially theorised by the study team helped to avoid limiting the scope of the interviews.^36^ Subsequent use of a data collection method sensitive to commercial pressures enabled the collection of information from a range of expert stakeholders. Stakeholders contributions transformed our understanding the marketing decision-making process, as illustrated in the contrast between the process we hypothesised before data collection (Supplementary file 2) and Figure 2 (developed after data collection).

Existing professional relationships between the study team and some interviewees increased sampling specificity but may have introduced confirmation bias. This was reduced by also including interviewees previously unknown to the interviewer in each of the three participant groups. Employing an alternative set of methods or interviewees may have led to different findings. Collecting data one year after the SDIL was implemented may have increased the credibility, with interviewees referencing marketing they knew to have occurred. However, this may have also introduced recall bias and limited expansive thinking. Focusing on the SDIL may have limited the transferability of findings to taxation policies designed differently.^15^ Exploring marketing responses to other stimuli, including sugary drinks taxes elsewhere, would determine the generalisability of the theoretical framework.

Establishing credibility of the specific concepts and processes in our final theoretical framework was helped by triangulation between researchers,^37^ and across a diverse set of stakeholder groups.^38^ Comparing the contribution from each interviewee group using an adapted convergence assessment highlighted differences in contribution rather than sentiment,^30^ suggesting that our study findings may resonate in other contexts. Our findings proved robust to validity testing using referential adequacy and member-checking,^34^ further increasing their credibility.

###  Comparison to Existing Literature

By employing a broad conceptualisation of marketing, this study has extended existing literature that implicitly or explicitly explores marketing changes following sugary drinks taxation. Changes to price and product composition following sugary drinks taxes have been reported for several countries,^19,39,40^ but often this is not explicitly considered in relation to other marketing activities that may influence the health outcomes of such taxes.^16,41^ The theoretical framework we developed is specific to sugary drinks taxes but echoes elements of existing marketing change models, such as the role of context and continuous market feedback,^42,43^ which increases the credibility of the model.

Our findings indicate that whether or not sugary drinks taxes lead to price increases, as has been documented elsewhere,^44^ will depend on individual company context. Decisions about price may be influenced by a company’s negotiation with retailers and their brand strength, partly explaining observed heterogeneity in the impact of the SDIL on prices. The framework emphasises that events like the SDIL are one of many factors considered when determining the form and nature of marketing. Reviewing their context in totality may result in a soft drinks company making either no changes to their marketing, changes that conflict or align with public health goals, or changes that are quickly augmented based on ‘feedback’ from sales. Emerging evidence confirms that some impacts of the SDIL, such as a reduction in soft drinks company profit,^20^ are small or short-lived, and may reflect responsive inward investment in marketing activities. Exploring circumstances leading to the alignment of commercial and public health goals could inform the design of sugary drinks taxation policies that are robust to industry resistance and counteraction.^45^ For example, our study highlighted that soft drinks companies were more likely to reformulate sugary drinks following the SDIL if they thought there was consumer interest in low sugar products and political appetite for further regulation.

###  Interpretation and Implications

Our theoretical framework proposes that using a broader concept of marketing leads to a better understanding of industry behaviour in response to public health policies such as sugary drinks taxes. This understanding could help develop integrated policy strategies that pre-empt and mitigate industry responses that undermine the public health goals of sugary drinks taxes. For example, our theoretical framework suggests a soft drinks company could offset any reduction in sales following a tax by changing their product packaging to appeal to consumers and retain sales, thus diluting any public health benefit of the price increase. Introducing mandatory labelling legislation alongside sugary drinks taxes, such as in Chile,^46^ could help avoid this.

The SDIL was specifically intended to encourage companies to reduce portion sizes and reformulate high sugar drinks to lower sugar alternatives.^17^ We found that factors that might drive a company to reformulate following taxation include their competitors’ reaction, consumers interests, previous positive experience of responding to policy, and a brand identity not tied to sugar. A company appeared more likely to reduce portion sizes if this was considered acceptable to consumers, they had high brand strength, access to the required infrastructure, and a brand identity tied to sugar. Though we explored these factors at a company-level, we also found evidence of sector-wide effects. For example, competitors’ reactions helped to normalise and perpetuate certain responses, making industry leaders particularly influential in determining the sector-wide response. Incentivising positive reactions from industry leaders may thus maximise the potential of future fiscal interventions.

The role of soft drinks companies’ external context in determining their marketing response to taxation suggests it may be possible to influence these environments to achieve particular responses. We found that a soft drinks company considers trends in policy implementation when reacting to a single policy like the SDIL. This suggests that integrated public health strategies may be more likely to elicit responses from industry aligned with policy-makers’ objectives than single interventions. Our finding that companies also determine their response based on concern for their reputation suggests that positioning a sugary drinks tax in the context of the benefits derived from its revenue (eg, hypothecation for children’s sport), might be another way that policy-makers can encourage certain responses from industry.^47^

###  Unanswered Questions and Future Work

Testing our framework with findings from the SDIL as well as those from other taxes on sugary drinks and less healthy foods will help clarify whether and how it is generalisable to other contexts.^48^ Such tests would benefit from data at a company- rather than industry-level, a broad conceptualisation of marketing and methods sensitive to temporal changes. Future work could also explore some of the factors we found may influence each company’s decisions, such as the role of retailers, in more detail.

The finding that reactions to sugary drinks taxes are likely to be company-specific suggests policy-makers could pay attention to individual- or groups of similar- companies, defined by characteristics like size or brand strength. Whilst it might be difficult to exhaustively assess the behaviour of every company, particular attention could be paid to market leaders. As the SDIL was already informed by industry consultation,^49^ it may be necessary for policy-makers to seek alternative avenues for understanding industry behaviour.

## Conclusion

 By employing a broad conceptualisation of marketing, we identified that soft drinks companies’ marketing reactions following the SDIL were heterogeneous and dependent on company-specific context. We illustrated that the process underpinning these decisions was continuous and iterative. Future work should explore what contexts can lead to marketing activities that enhance or undermine public health aims of sugary drinks taxes, to inform the design of future fiscal interventions. Doing so might have more general relevance for understanding how the food and drinks industry responds to a wider range of public health regulatory interventions, and the consequences for achieving public health gain.

## Acknowledgements

 The authors would like to thank interviewees who agreed to take part in this study.

## Ethical issues

 A favourable opinion on the study was obtained from the University of Cambridge Humanities and Social Science Research Ethics Committee (approved 16 May 2018, ref: 18/165).

## Competing interests

 HF reports PhD funding from Public Health England and from Economic and Social Research Council, grants from Murray Edwards College, Cambridge; personal fees from University of Cambridge School of Clinical Medicine, personal fees from Cambridge Social Science Partnership, outside the submitted work; and HF has previously worked for market research companies which conduct research on behalf of many companies, including those from the food and drink industry. LL is a Visiting Professor at the University of Chester. FG is now employed by The National Institute for Health and Care Excellence.

## Authors’ contributions

 All authors attest they meet the ICMJE criteria for authorship. Conception and design: HF, TP, MW, LL, FG, and JA; acquisition of data: HF; analysis and interpretation of data: HF, TP, MW, LL, FG, and JA; drafting of the manuscript: HF; critical revision of the manuscript for important intellectual content: TP, MW, LL, FG, and JA; obtaining funding: MW, LL, FG, and JA; administrative, technical or material support: HF; supervision: MW, LL, FG, and JA.

## Funding

 HF received funding for her PhD studentship from the Economic and Social Research Council and Public Health England, and she has received further discretionary funding from the Economic and Social Research Council and Murray Edwards College, Cambridge. During the course of this work, HF, JA and MW were supported by the Medical Research Council (grant number MC_UU_00006/7). MW received funding as Director of NIHR’s Public Health Research Programme. FG and LL were employed by Public Health England at the time the study was conducted. MW holds an honorary consultant in public health contract with Public Health England, and JA holds an honorary contract with Public Health England. Public Health England, a government-funded arm’s length body and one of the funders, could have influenced study design, data collection, data analysis, data interpretation and writing of the manuscript via the involvement of FG and LL and informal feedback received at seminars where this work was presented. No other funders had any influence.

## Supplementary files


Supplementary file 1. COREQ (COnsolidated criteria for REporting Qualitative research) Checklist.

Supplementary file 2. Pre-interview Material.

Supplementary file 3. Interview Guide.

Supplementary file 4. Summary of Findings.
