# Supplementary material for: Understanding Marketing Responses to a Tax on Sugary Drinks: A Qualitative Interview Study in the United Kingdom, 2019
Source: Int J Health Policy Manag. 2022 Feb 23;11(11):2618–29. doi: 10.34172/ijhpm.2022.5465 (PMC9818127; doi:10.34172/ijhpm.2022.5465)
Supplement: Supplementary file 1 — COREQ (COnsolidated criteria for REporting Qualitative research) Checklist. [file ijhpm-11-2618-s001.pdf]

Article title: Understanding Marketing Responses to a Tax on Sugary Drinks: A Qualitative Interview Study in the United Kingdom, 2019

Journal name: International Journal of Health Policy and Management (IJHPM)

Authors' information: Hannah Forde[1]*, Tarra L. Penney[2,1], Martin White1, Louis Levy[3,4], Felix Greaves[5,6,4], Jean Adams[1]

[1]MRC Epidemiology Unit, University of Cambridge School of Clinical Medicine, Institute of Metabolic Science, Cambridge, UK.

[2]Global Food Systems and Policy Research, School of Global Health, Faculty of Health, York University, Toronto, ON, Canada.

[3]The Faculty of Health and Social Care, University of Chester, Chester, UK.

[4]Public Health England, London, UK.

[5]Department of Primary Care and Public Health, School of Public Health, Imperial College London, London, UK. 6National Institute for Health and Care Excellence, London, UK.

(*Corresponding author: hf332@medschl.cam.ac.uk)

Supplementary file 1. Coreq (Consolidated Criteria For Reporting Qualitative Research) Checklist.

**Topic**	**Item no.**	**Guide question/description**	**Page**
**Domain 1: Research team and reflexivity**			
Personal characteristics			
Interviewer/facilitator	1	Which author/s conducted the interview or focus group?	3
Credentials	2	What were the researcher's credentials? E.g. PhD, MD	Title page
Occupation	3	What was their occupation at the time of study?	Title page
Gender	4	Was the researcher male or female?	N/A
Experience and training	5	What experience or training did the researcher have?	4
Relationship with participants			
Relationship established	6	Was a relationship established prior to study commencement?	3
Participant knowledge of the interviewer	7	What did the participants know about the researcher? e.g. personal goals, reasons for doing the research	3
Interviewer characteristics	8	What characteristics were reported about the interviewer/facilitator? E.g. bias, assumptions, reasons and interests in the research topic	3

**Domain 2: Study design**			
Theoretical framework			
Methodological orientation and Theory	9	What methodological orientation was stated to underpin the study? e.g. grounded theory, discourse analysis, ethnography, phenomenology, content analysis	2
Participant selection			
Sampling	10	How were participants selected? e.g. purposive, convenience, consecutive, snowball	2
Method of approach	11	How were participants approached? e.g. face-to-face, telephone, mail, email	2
Sample size	12	How many participants were in the study?	4
Non-participation	13	How many participants refused to participate or dropped out? Reasons?	4
Setting			
Setting of data collection	14	Where was the data collected? e.g. home, clinic, workplace	3
Presence of non-participants	15	Was anyone else present besides the participants and researchers?	3
Description of sample	16	What are the important characteristics of the sample? e.g. demographic, data, date	4
Data collection			
Interview guide	17	Were questions, prompts, guides provided by the authors? Was it pilot tested?	3
Repeat interviews	18	Were repeat interviews carried out? If yes, how many?	N/A
Audio/visual recording	19	Did the research use audio or visual recording to collect the data?	3
Field notes	20	Were field notes made during and/or after the interview or focus group?	3
Duration	21	What was the duration of the interviews or focus groups?	4
Data saturation	22	Was data saturation discussed?	2
Transcripts returned	23	Were transcriptions returned to participants for comment and/or correction?	4
**Domain 3: analysis and findings**			
Data analysis			
Number of data coders	24	How many data coders coded the data?	4
Description of the coding tree	25	Did authors provide description of the coding tree?	5

Derivation of themes	26	Were themes identified in advance or derived from the data?	3-4
Software	27	What software, if applicable, was used to manage the data?	4
Participant checking	28	Did participants provide feedback on the findings?	4
Reporting			
Quotations presented	29	Were participant quotations presented to illustrate the themes/findings? Was each quotation identified? e.g. participant number	5
Data and findings consistent	30	Was there consistency between the data presented and the findings?	5-8
Clarity of major themes	31	Were major themes clearly presented in the findings?	5-8
Clarity of minor themes	32	Is there a description of diverse cases or discussion of minor themes?	8

Developed from: Tong A, Sainsbury P, Craig J. Consolidated criteria for reporting qualitative research (COREQ): a 32-item checklist for interviews and focus groups. International Journal for Quality in Health Care. 2007. Volume 19, Number 6: pp. 349 – 357
