# Supplementary material for: Understanding Marketing Responses to a Tax on Sugary Drinks: A Qualitative Interview Study in the United Kingdom, 2019
Source: Int J Health Policy Manag. 2022 Feb 23;11(11):2618–29. doi: 10.34172/ijhpm.2022.5465 (PMC9818127; doi:10.34172/ijhpm.2022.5465)
Supplement: Supplementary file 2 — Pre-interview Material. [file ijhpm-11-2618-s002.pdf]

Article title: Understanding Marketing Responses to a Tax on Sugary Drinks: A Qualitative Interview Study in the United Kingdom, 2019

Journal name: International Journal of Health Policy and Management (IJHPM)

Authors' information: Hannah Forde[1]*, Tarra L. Penney[2,1], Martin White1, Louis Levy[3,4], Felix Greaves[5,6,4], Jean Adams[1]

[1]MRC Epidemiology Unit, University of Cambridge School of Clinical Medicine, Institute of Metabolic Science, Cambridge, UK.

[2]Global Food Systems and Policy Research, School of Global Health, Faculty of Health, York University, Toronto, ON, Canada.

[3]The Faculty of Health and Social Care, University of Chester, Chester, UK.

[4]Public Health England, London, UK.

[5]Department of Primary Care and Public Health, School of Public Health, Imperial College London, London, UK. 6National Institute for Health and Care Excellence, London, UK.

(*Corresponding author: hf332@medschl.cam.ac.uk)

Supplementary file 1. Pre-interview Material

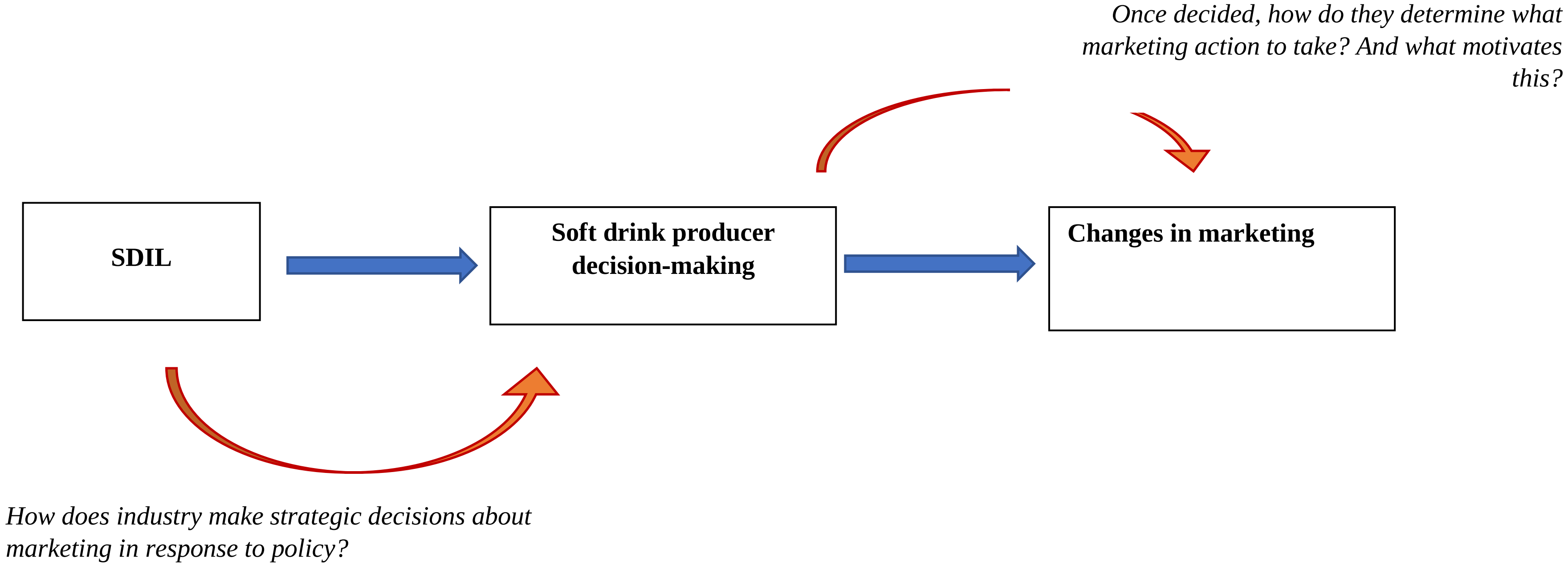

Framework narrative

The introduction of the UK Soft Drinks Industry Levy (SDIL) is intended to help kerb the heavy consumption of sugar in the UK that currently contributes towards a number of non-communicable diseases. However, the effect it has on the purchasing and consumption of soft drinks will depend on how various stakeholders choose to react. It is expected that one of the many responses to the SDIL will involve soft drinks producers changing the marketing of their products, but it is unclear from existing research exactly how and why these changes will manifest.

This framework summarises some of the possible stages of how and why soft drink companies may change the marketing of their products in response to the SDIL. We anticipate the decision-making process and determining specific marketing actions will be a complex process involving many factors. Broadly we are expecting that industry makes decisions about policies, like the SDIL, that might affect their profits and reputation; and then they determine exactly what action to take in relation to marketing. To help us understand how this might work, we would like to collect any insights you would be willing to share relating to any aspect of this framework.
