# Supplementary material for: Understanding Marketing Responses to a Tax on Sugary Drinks: A Qualitative Interview Study in the United Kingdom, 2019
Source: Int J Health Policy Manag. 2022 Feb 23;11(11):2618–29. doi: 10.34172/ijhpm.2022.5465 (PMC9818127; doi:10.34172/ijhpm.2022.5465)
Supplement: Supplementary file 3 — Interview Guide. [file ijhpm-11-2618-s003.pdf]

Article title: Understanding Marketing Responses to a Tax on Sugary Drinks: A Qualitative Interview Study in the United Kingdom, 2019

Journal name: International Journal of Health Policy and Management (IJHPM)

Authors' information: Hannah Forde[1]*, Tarra L. Penney[2,1], Martin White1, Louis Levy[3,4], Felix Greaves[5,6,4], Jean Adams[1]

[1]MRC Epidemiology Unit, University of Cambridge School of Clinical Medicine, Institute of Metabolic Science, Cambridge, UK.

[2]Global Food Systems and Policy Research, School of Global Health, Faculty of Health, York University, Toronto, ON, Canada.

[3]The Faculty of Health and Social Care, University of Chester, Chester, UK.

[4]Public Health England, London, UK.

[5]Department of Primary Care and Public Health, School of Public Health, Imperial College London, London, UK. 6National Institute for Health and Care Excellence, London, UK.

(*Corresponding author: hf332@medschl.cam.ac.uk)

Supplementary file 3. Interview Guide

<u>Note:</u> Changes made following pilot interviews are marked in red

**Icebreaker – Your role and organisation**		
**Check**	**Question**	**Prompt/probe**
	Position, title and organisation, length of time in position	• What is the main role/objective of your organisation?<br>• What are the objectives of your role within the organisation?<br>• What are your main responsibilities?
	~~**[IF NECESSARY]** Area of specialist interest/role~~	~~• What are the objectives of your role within the organisation?~~<br>~~• What are your main responsibilities?~~
**Section 1 – Understanding of the SDIL**		
**Check**	**Question**	**Prompt/probe**
	As we discussed, in April ~~this~~ last year the UK introduced a soft drinks industry levy (sometimes referred to as the "sugar tax").	
	Before this interview, had you heard of the Levy?<br><br>[IF YES] What are your thoughts on the Levy? **[AC]**	
	[Adapt according to response] To confirm, the soft drinks industry levy, or "sugar tax" was announced two years prior to its introduction, in the Chancellor of the Exchequer's budget speech of 2016. In part, this was to enable soft drinks industries to respond to this measure by various means. The levy is designed to encourage manufacturers to reduce the sugar content of their drinks, and reduce sugar consumption, in light of increasing evidence association heavy sugar intake with numerous non-communicable diseases.	
**Section 2 – Industry response to the SDIL**		
**Check**	**Question**	**Prompt/probe**
	In general, how do you think industry make decisions about policies that might affect their profits or reputation?	• What factors do they consider when making these decisions?<br>• Who are the stakeholders in these decisions?
	What do you think soft drink company's first response to the SDIL may have been?	• Did you hear reports of their response or their reaction at the time?<br>• If so, where?

	Policymakers and public health specialists think that the levy is likely to have various effects, and that these will vary for different groups of people. In part, this will depend on how the main stakeholders affected by the levy choose to respond. One way soft drink producers might respond is by changing the way soft drinks are marketed to consumers. **We would like to 'map' the various decision processes and industry strategies that might explain changes in marketing, and have created a framework to initiate this process [SHOW].**	
	Firstly, how would you define marketing?	
	What is your understanding of food and drink marketing?	• How do you think food and drink is commonly marketed?<br>• ~~What do you think are the effects of food and drink marketing?~~<br>• Among which audiences/populations/consumer groups do you think marketing might be most effective?<br>• Do you think marketing has a positive or negative influence on an individual's ability to maintain a healthy lifestyle?
	[Adapt/If necessary] For this interview, I would like you to think about soft drink marketing as any communication that soft drink companies may have with their potential consumers. This could be include direct marketing, through television or social media advertising, to less obvious communication such as sponsorship or product placement.	
**Section 3 – Motivation to change marketing**		
**Check**	**Question**	**Prompt/probe**
	To what extent do you agree that soft drink marketing is likely to change in response to the levy?	• Explain [adapt subsequent questions accordingly]
	What do you think are the main factors motivating soft drink producers to respond to the tax using marketing?	• Aside from the levy, what else do you think producers may have considered when deciding if/how to respond?
	Which stakeholders are involved in the decision of if/how to respond to the levy through marketing?	• Internal/external?<br>• Relative importance?
**Section 4 – Manifestation of changes to marketing**		
**Check**	**Question**	**Prompt/probe**
	There are a variety of ways in which food and drinks products are marketed.	
	• What specific changes to marketing are likely to arise as a result of the tax?	• Will these relate to the message? Nature? Medium? Scale? Audience?<br>• Do you think these changes will take place in any particular order

	• How will industry decide what changes to make?	• What factors are likely to affect their decision?
	• How effective do you think these changes will be in attaining the goals of the soft drinks producers?	• Explain
**Concluding remarks and future research**		
	That is the end of the questions I have for you. [Summarise discussion] Do you have any other thoughts or anything that you would like to say about the issues discussed today, or that I haven't asked you about? Would you like a summary of the findings when available?<br><br>I will email you in approximately two-three months for you to provide feedback on the systems map. It is likely that later in my PhD project, I will conduct more interviews to learn more about the soft drinks industry levy. Would you be interested in being contacted for future research? Thank you for taking part.<br><br>[RECORDER OFF] Ask for snowball sampling	
