# Supplementary material for: Understanding Marketing Responses to a Tax on Sugary Drinks: A Qualitative Interview Study in the United Kingdom, 2019
Source: Int J Health Policy Manag. 2022 Feb 23;11(11):2618–29. doi: 10.34172/ijhpm.2022.5465 (PMC9818127; doi:10.34172/ijhpm.2022.5465)
Supplement: Supplementary file 4 — Summary of Findings. [file ijhpm-11-2618-s004.pdf]

Changes to soft drinks marketing after taxation

Developing a general theory

CEDAR
Centre for Diet and Activity Research
www.cedar.iph.cam.ac.uk

Hannah Forde, PhD Student, hf332@medschl.cam.ac.uk

October 2019

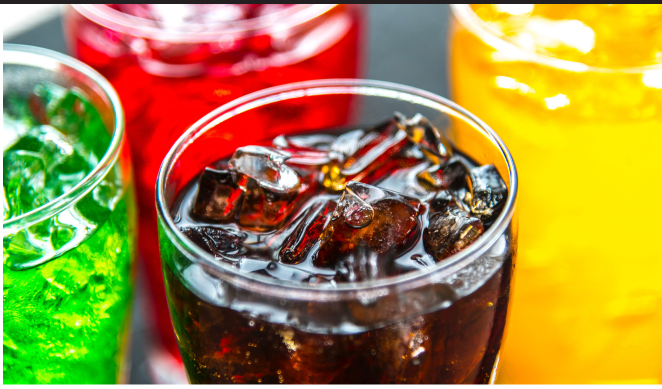

Earlier this year, you contributed to research about soft drinks marketing following introduction of the UK Soft Drinks Industry Levy (SDIL). We have used participants' contributions to develop a general theory outlining how the marketing of soft drinks might change in response to the SDIL.

Now we need your help! Please read this briefing and respond to the questions in the covering email.

Taxing sugary drinks is an increasingly popular way to try and reduce sugar consumption. In the UK, the Levy (SDIL) varies by the sugar content of drinks in order to encourage soft drink producers to reformulate their drinks.

However in order to avoid losing profit, soft drink producers may also respond to the Levy by changing the marketing of their products. As there is very little existing evidence to illustrate how marketing may change, we aimed to develop a general theory – a basic articulation for how and why things happen – to help understand the role of different stakeholders and context in processes of change.

To achieve this, we built an **initial theory** that we shared with you earlier this year:

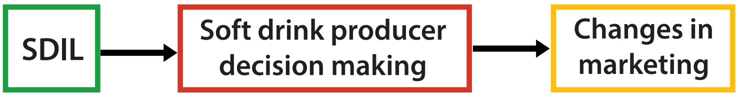

We then conducted **18 telephone interviews** with members of industry, academia and civil society, and **analysed interview transcripts** to elaborate and develop the initial theory.

The developed theory. A visualisation is shown below, with further explanation overleaf.

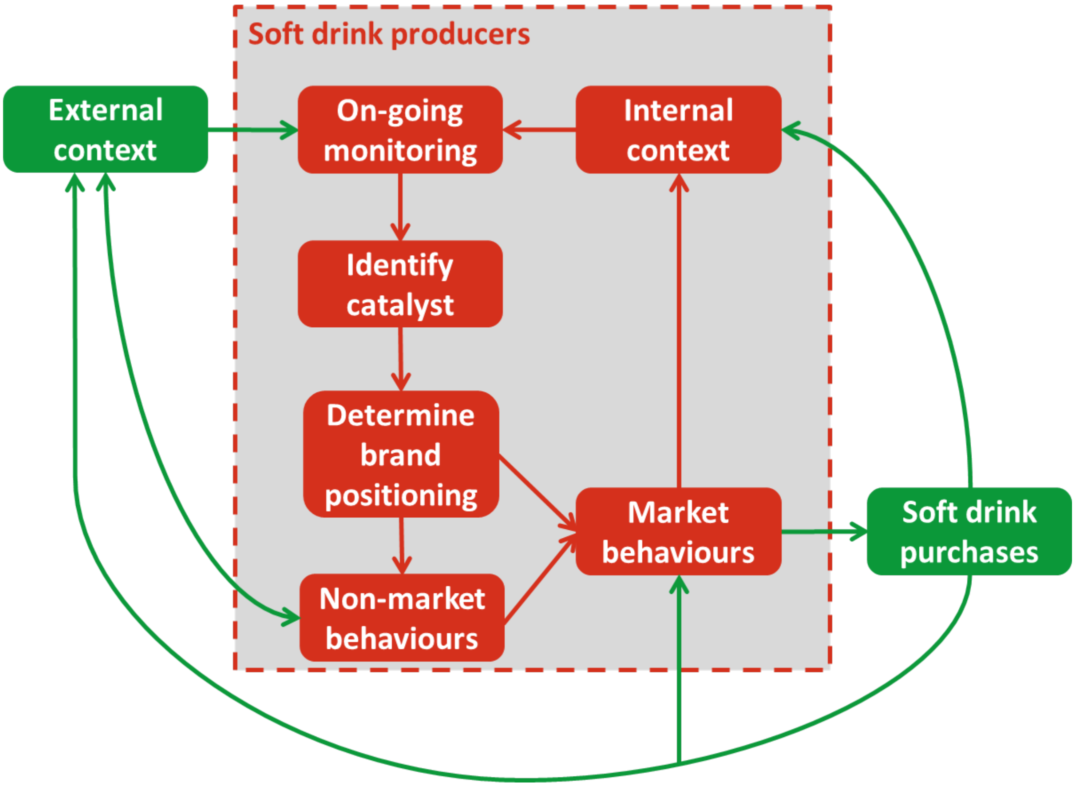

Processes of marketing change proposed in the theory

Your insight led us to the following observations.

- **Soft drink producers** continuously monitor their internal and external context, as this affects a firm's decision to change marketing and the options available to them.
- **Internal factors** for producers include brand portfolio and brand strength, human and physical capital, values. **External factors** include consumer trends, retailer activity, and other government policies.
- A culmination of trends observed in these contexts combined with a **catalyst**, like the SDIL, means a firm decides to **reposition their soft drink brand.** Brand repositioning could involve a combination of various **market and non-market behaviours.** These behaviours are co-ordinated and strategically selected.
- **Market behaviours** are those exhibited to the consumer, like reformulation, acquiring products from elsewhere, developing new products altogether, changing a product's price or packaging.
- **Non-market behaviours** do not directly affect consumers, but work to change the context of a soft drinks firm. Lobbying and using the media are examples of non-market behaviours.
- A combination of these market and non-market behaviours are intended to affect **soft drink purchases**, which in turn affects the internal and external context of a soft drinks firm, thus creating a cycle of marketing change.

Key characteristics of the developed theory

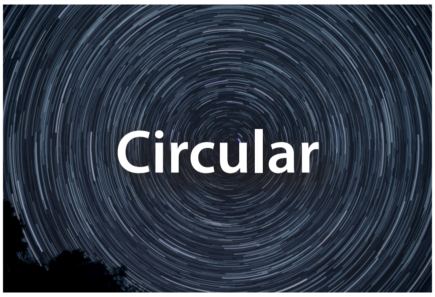

The initial theory described a linear change process of strategic planning followed by specific marketing responses.

Analysing interviewee contributions led us to believe that changes to marketing are actually circular and iterative.

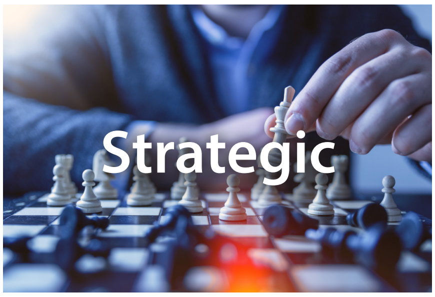

There are a variety of ways that producers may change marketing in response to soft drink taxation.

The theory highlights the strategy and co-ordination underlying changes to soft drink producers' market and non-market behaviours.

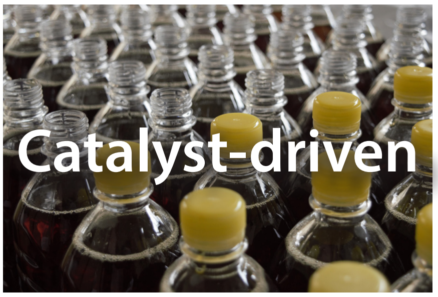

The theory proposes that soft drink firms continuously monitor factors internal and external to their organisation.

Identifying a catalyst like the SDIL in their internal or external context accelerates existing evolution of changes to marketing.

Now we need your help!

In the final stage of this research, we want to check that the theory we have developed is an accurate reflection of your experiences of soft drinks marketing.

To do this, please answer by email to **hf332@medschl.cam.ac.uk** by **24 October 2019**. Please also get in touch if you have any questions about this stage of your involvement in the research.

This research is being carried out by the University of Cambridge, and has been approved by the Humanities and Social Sciences Research Ethics Committee. If you have any questions, complaints or reservations about your participation in this study which you cannot resolve with the research lead (**hf332@medschl.cam.ac.uk**), you may contact the ethic committee directly at **cshssoffice@admin.cam.ac.uk**, quoting reference **18/165**. Any complains or enquiry will be treated in confidence and investigated fully.
